# Subclinical atherosclerosis and risk factors in relation to autonomic indices in the general population

**DOI:** 10.1097/HJH.0000000000003397

**Published:** 2023-03-20

**Authors:** Christian Zambach, Artur Fedorowski, Sofia Gerward, Madeleine Johansson, Gunnar Engström, Viktor Hamrefors

**Affiliations:** a Department of Clinical Sciences, Lund University, Malmö; b Department of Internal Medicine, Skåne University Hospital, Lund; c Department of Cardiology, Karolinska University Hospital, and Department of Medicine, Karolinska Institutet, Stockholm; d Department of Cardiology, Skåne University Hospital, Malmö, Sweden

**Keywords:** arterial stiffness, autonomic cardiovascular dysfunction, cardiovascular risk factors, coronary artery calcification score, coronary artery calcium score, orthostatic hypotension, orthostatic reactions, resting heart rate

## Abstract

Orthostatic hypotension and resting heart rate (RHR) are associated with cardiovascular disease (CVD). However, it is unknown how these factors relate to subclinical CVD. We examined the relationship between orthostatic blood pressure (BP) response, RHR and cardiovascular risk factors, including coronary artery calcification score (CACS) and arterial stiffness, in the general population.

**Methods:**

We included 5493 individuals (age 50–64 years; 46.6% men) from The Swedish CArdioPulmonary-bio-Image Study (SCAPIS). Anthropometric and haemodynamic data, biochemistry, CACS and carotid-femoral pulse wave velocity (PWV) were retrieved. Individuals were categorized into binary variables that manifest orthostatic hypotension and in quartiles of orthostatic BP responses and RHR, respectively. Differences across the various characteristics were tested using χ^2^ for categorical variables and analysis of variance and Kruskal–Wallis test for continuous variables.

**Results:**

The mean (SD) SBP and DBP decrease upon standing was -3.8 (10.2) and -9.5 (6.4) mmHg, respectively. Manifest orthostatic hypotension (1.7% of the population) associated with age (*P* = 0.021), systolic, diastolic and pulse pressure (*P* < 0.001), CACS (<0.001), PWV (*P* = 0.004), HbA1c (*P* < 0.001) and glucose levels (*P* = 0.035). Age (*P* < 0.001), CACS (*P* = 0.045) and PWV (*P* < 0.001) differed according to systolic orthostatic BP, with the highest values seen in those with highest and lowest systolic orthostatic BP-responses. RHR was associated with PWV (*P* < 0.001), SBP and DBP (*P* < 0.001) as well as anthropometric parameters (*P* < 0.001) but not CACS (*P* = 0.137).

**Conclusion:**

Subclinical abnormalities in cardiovascular autonomic function, such as impaired and exaggerated orthostatic BP response and increased resting heart rate, are associated with markers of increased cardiovascular risk in the general population.

## INTRODUCTION

Orthostatic hypotension is one common manifestation of cardiovascular autonomic dysfunction that affects a substantial proportion of the population [1]. The incidence of orthostatic hypotension increases with age and the presence of orthostatic hypotension is associated with neurologic disorders as well as cardiovascular risk factors and disease [2,3]. Apart from the adverse outcomes of manifest orthostatic hypotension and the morbidity and mortality in conjunction with fall injuries [4], even subtle indices of autonomic dysfunction can be the first signs of disease and predict future adverse cardiovascular events [1–3,5,6]. This may be in the form of an abnormal orthostatic blood pressure response that does not fulfil the criteria for manifest orthostatic hypotension, but cardiovascular autonomic dysfunction may also be indicated by an elevated resting heart rate (RHR). Of note, an elevated RHR has been shown to be strongly associated with adverse cardiovascular events [6,7].

Whereas markers of cardiovascular autonomic dysfunction, such as orthostatic hypotension and elevated RHR, are associated with adverse cardiovascular outcomes, it is not known how these factors relate to subclinical cardiovascular disease in the general population. One measurement of vascular ageing and adverse cardiovascular risk is arterial stiffness [8,9]. With increasing age, the walls of large elastic arteries undergo adverse structural and functional changes, leading to arteriosclerosis and arterial stiffness [10]. Arterial stiffness can be determined by various indices, of which carotid to femoral pulse wave velocity (PWV) is the most widely validated [11].

Another potential marker of vascular ageing and subclinical atherosclerosis is coronary artery calcification score (CACS) according to Agatson, which is assessed by computed tomography (CT). Recent studies have suggested that CACS is of predictive value, particularly in asymptomatic individuals [12–16] and may reclassify individuals at risk of coronary artery disease events [17].

In this study, we examined signs of cardiovascular autonomic dysfunction in the general population and their association with cardiovascular risk factors including subclinical atherosclerosis and arterial stiffness. We specifically hypothesized that an abnormal orthostatic blood pressure response as well as elevated RHR in middle-aged individuals are associated with subclinical cardiovascular disease in the form of increased arterial stiffness, coronary and carotid atherosclerosis.

## MATERIALS AND METHODS

### Study population

The current study was based on the population-based Swedish CArdioPulmonary-bio-Image Study (SCAPIS) from which we used data from the sub-cohort recruited in Malmö, Sweden. The Malmö SCAPIS cohort (6251 individuals, 53% participation rate of invited individuals) included blood samples, questionnaires, anthropometric and haemodynamic measurements, including complete recordings on orthostatic blood pressure reactions, coronary artery calcium score as well as carotid to femoral PWV. The SCAPIS project has been previously described in detail [18,19].

**FIGURE 1:**
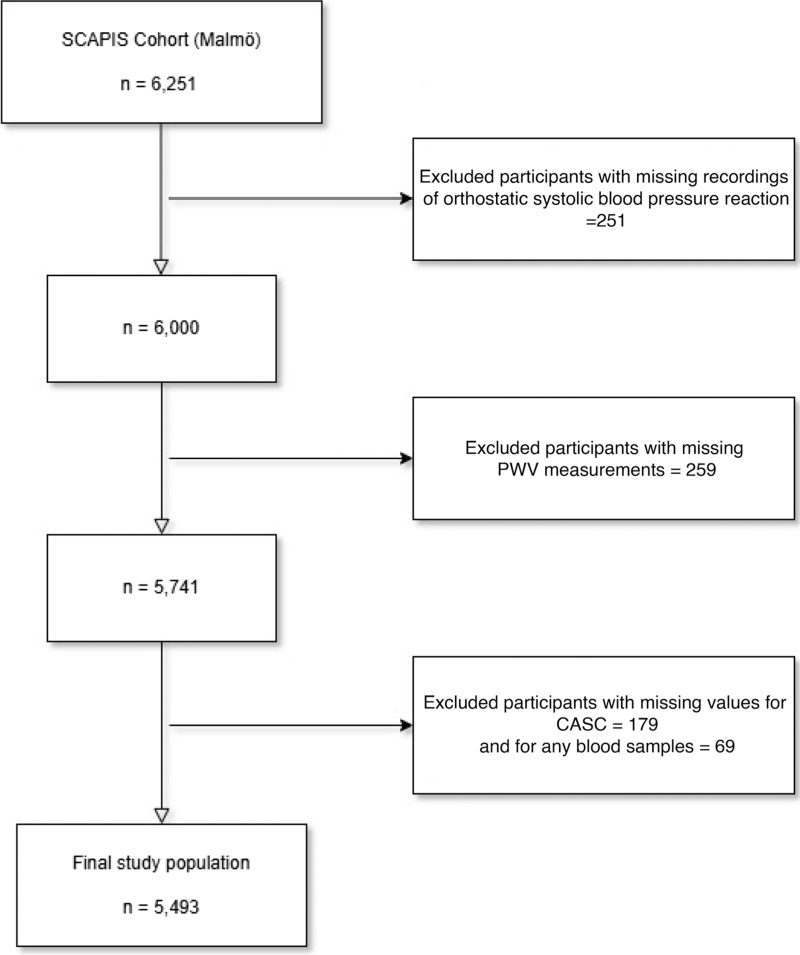
Flow chart for the selection of the final study population. The selection of the final study population. PWV, pulse wave velocity; SCAPIS, The Swedish CArdioPulmonary bioImage Study.

Figure 1 depicts the procedure of exclusion in our current study population. Firstly, individuals with missing recordings of orthostatic blood pressure (BP) reactions (*n* = 251) were excluded. Of the remaining 6000 individuals, 259 were excluded due to missing information on carotid-femoral PWV. Of these, 5741 individuals, 179 were further excluded due to missing information on CACS and 69 more for missing information of any blood samples. In total, 5493 individuals constituted the final study population. These individuals had complete data on anthropometric measurements, that is weight, height, BMI and waist circumference, smoking status, BP and pulse pressure (PP) and RHR.

Written informed consent was obtained from all participants. The SCAPIS project was approved by the Regional Ethical Review Board in Umeå (2010–228–31 M) and Lund (2016/1031), respectively.

### Basic examination

Computed tomography was used to determine CACS, and the calcium content in each artery was summed utilizing the Agatston scoring system [20,21].

Arterial stiffness was determined by the parameter carotid-femoral artery PWV in metres per second. The methods for assessing CACS and PWV in the SCAPIS study were described in detail previously [18]. Height, weight, waist circumference and BMI were obtained and measured in meters and kilograms.

Brachial SBP and DBP readings were taken in supine position after 5 min of rest. BP readings were then taken after 3 min of standing to obtain systolic and diastolic orthostatic BP reactions by subtracting the BP after standing from the BP in supine position (i.e. positive values correspond to a BP decrease on standing).

Orthostatic hypotension was defined according to the international consensus as a decrease in SBP of 20 mmHg or more and/or a decrease in DBP of 10 mmHg or more. We also created another variable for orthostatic hypotension that included a drop in SBP to below 90 mmHg 3 min after standing up (OH90). Fasting venous blood samples were collected for analysis of glycated haemoglobin (HbA1c), plasma lipids, creatinine and CRP. Smoking and antihypertensive drug treatment were assessed from self-reported data from a questionnaire. Smoking status was categorized as current smoker, former smoker and never-smoker, respectively. Pack-years of smoking was calculated for current smokers (*n* = 813) and former smokers. Individuals were classified as having diabetes based on responses in the questionnaire or by an elevated fasting plasma glucose sample. Detailed information on the methods used in the Malmö cohort of SCAPIS are found elsewhere [18,19].

### Statistical analysis

**FIGURE 2:**
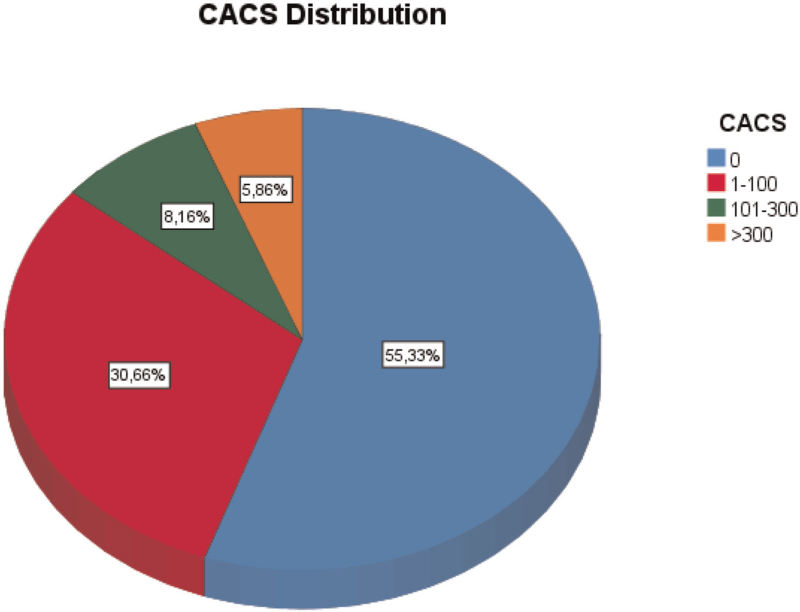
Distribution of coronary artery calcification score in the study population. Distribution of coronary artery calcification score in the study population. The score corresponds to Agatson score measured by computed tomography. CACS, coronary artery calcification score.

More than 50% of the participants had a calcium score of zero, resulting in skewed data. To classify the distribution (Fig. 2), CACS was classified into four categories using cut offs: 0, 1–100, 101–300 and more than 300.

The participants were categorized into quartiles according to orthostatic BP decrease, that is Q1 included the individuals with the least decrease (or even increase in BP upon standing) and Q4 those with the most fall in BP upon standing.

The characteristics of the study population were explored and described as mean ± SD for normal distributions. Due to skewed distribution, CACS was described as median and interquartile range.

Differences across the various characteristics were tested using χ^2^ for categorical variables, analysis of variance (ANOVA) for continuous and normally distributed variables and Kruskal–Wallis test and Mann–Whitney test for nonnormal-distributed variables. Furthermore, linear regression models were constructed with CACS and PWV, respectively, as dependent variables and orthostatic hypotension as well as covariates age, sex, smoking, diabetes mellitus, low-density lipoprotein (LDL) and antihypertensive treatment as independent variables. All analyses were carried out using SPSS version 25 (IBM Corp, Armonk, New York, USA). A *P* value of less than 0.05 was regarded as statistically significant.

## RESULTS

### Characteristics of the study population

Characteristics of the study population are presented in Table 1. In short, the participants were between 50 and 64 years of age, and 46.6% of participants were men. In the population, 43% were overweight (BMI greater than 25 kg/m^2^) and 22% were obese (BMI greater than 30 kg/m^2^). This distribution is depicted in a pie chart in the supplement (Supplementary figure 1). Diabetes mellitus was prevalent in 7.7%. The mean (SD) orthostatic SBP and DPB reactions upon standing was −3.8 (10.2) and −9.5 (6.4) mmHg, respectively (i.e. both SBP and DBP increased on average in the population). Manifest orthostatic hypotension was present in 95 of 5493 recordings, that is 1.7% and 124, that is 2.3% when also accounting for a drop of SBP to under 90 mmHg (OH90).

**TABLE 1:** Study population characteristics

		Orthostatic SBP reaction				
Characteristics	*N*	Q1 (Increase > 10 mmHg)	Q2 (Increase 10 to 4 mmHg)	Q3 (Increase 4 mmHg to decrease 3 mmHg)	Q4 (Decrease > 3 mmHg)	*P* ^b^
Number of individuals (*n*, %)	5493	1478 (26.9%)	1306 (23.8%)	1363 (24.8%)	1346 (24.5%)	0.000
Age (years)	57.5 (±4.3)	58 (±4.3)	57.1 (±4.3)	57.1 (±4.3)	57.6 (±4.3)	0.000
Male sex (*n*, %)	2561 (46.6%)	42.8%	45.3%	48.7%	50%	0.000
BMI (kg/m^2^)	27 (±4.3)	27.5 (±4.6)	26.9 (±4.2)	26.8 (±4.3)	26.8 (±4.2)	0.000
Weight (kg)	79.9 (±15.7)	80 (±15.9)	79.5 (±15.4)	79.8 (±16.9)	80.3 (±15.7)	0.570
Height (cm)	171.6 (±9.7)	170.3 (±9.6)	171.5 (±9.6)	172.1 (±9.8)	172.9 (±9.7)	0.000
Waist circumference (cm)	94.8 (±12.7)	95.7 (±12.8)	94.5 (±12.5)	94.4 (±12.7)	94.7 (±12.7)	0.019
Resting heart rate (beats per minute)	61 (±9)	61 (±9)	61 (±9)	61 (±9)	61 (±9)	0.743
SBP (mmHg)	123 (±16)	121 (±16)	120 (±16)	122 (±16)	128 (±17)	0.000
DBP (mmHg)	76 (±10)	76 (±9)	76 (±10)	76 (±10)	78 (±10)	0.000
Pulse pressure (mmHg)	46 (±10)	45 (±10)	44 (±9)	46 (±9)	50 (±10)	0.000
Carotid plaque (%)	3278 (59.9%)	59.3%	56.6%	60.6%	62.8%	0.012
Coronary artery calcium score^a^	0 (28)	0 (30)	0 (23)	0 (27)	0 (32)	0.045
Pulse wave velocity (m/s)	8.34 (±1.26)	8.36 (±1.29)	8.19 (±1.19)	8.28 (±1.2)	8.53 (±1.31)	0.000
Pack-years	27 (±34)	28 (±36)	27 (±31)	28 (±34)	26 (±32)	0.310
Haemoglobin level (g/dl)	143 (±12)	142 (±12)	142 (±12)	143 (±12)	143 (±12)	0.091
Creatinine level (mmol/l)	77 (±15)	77 (±16)	77 (±15)	78 (±15)	78 (±15)	0.030
Triglyceride level (mg/dl)	1.3 (±0.8)	1.3 (±0.8)	1.3 (±0.8)	1.2 (±0.8)	1.2 (±0.7)	0.213
Cholesterol level (mg/dl)	5.5 (±1)	5.5 (±1)	5.5 (±1)	5.4 (±1)	5.4 (±1)	0.009
HDL (mmol/l)	1.7 (±0.5)	1.7 (±0.5)	1.7 (±0.5)	1.7 (±0.5)	1.7 (±0.5)	0.441
LDL (mmol/l)	3.6 (±0.9)	3.6 (±0.9)	3.6 (±1)	3.6 (±0.9)	3.6 (±1)	0.068
Cholesterol/HDL ratio	3.6 (±1.3)	3.6 (±1.4)	3.6 (±1.2)	3.5 (±1.2)	3.6 (±1.2)	0.424
CRP (mg/l)	2.4 (±4.4)	2.6 (±4.9)	2.2 (±3.8)	2.4 (±5.4)	2.1 (±3.1)	0.012
HbA1c (%)	36.9 (±6.8)	36.8 (±5.9)	36.7 (±5.8)	36.8 (±6.6)	37.3 (±8.8)	0.150
Plasma glucose level (mmol/l)	5.5 (±1.2)	5.5 (±1.1)	5.5 (±1.2)	5.5 (±1.1)	5.6 (±1.5)	0.014
Diabetes (*n*, %)	423 (7.7%)	8.4%	6.2%	7.5%	8.6%	0.079
Current smokers (*n*, %)	813 (14.8%)	15.9%	16.6%	14.3%	12.3%	0.009
Antihypertensive drugs (*n*, %)	1100 (20%)	23.8%	17.5%	19.3%	19%	0.001

Values expressed are means (±SD) or percentages unless specified otherwise. CRP, C-reactive protein; HDL, high-density lipoprotein cholesterol; LDL, low-density lipoprotein cholesterol.

a Expressed as median (third quartile) due to skewed distribution.

b *P* value is for difference across the quartiles of Delta SBP.

The distribution of the CACS in the population is depicted in Fig. 2. Approximately 55% had no signs of coronary artery calcification on cardiac computerized tomography, while about 6% had severe calcification of their coronary arteries (CACS >300).

Measurements on carotid-femoral PWV in our study (mean 8.34 m/s; SD 1.26) resembled findings for the same age group found in other studies [22,23]. Haemodynamic parameters and blood samples had normal values on average.

### Cardiovascular risk factors and orthostatic hypotension

The distribution of cardiovascular risk factors in relation to manifest orthostatic hypotension is depicted in Table 2. The factors that differed according to orthostatic hypotension were age (*P* = 0.021), SBP (*P* < 0.001), DBP (*P* < 0.001), PP (*P* < 0.001), CACS (*P* < 0.001), PWV (*P* = 0.004) as well as HbA1c (*P* < 0.001) and plasma glucose levels (*P* = 0.035). The occurrence of carotid plaque did not differ according to orthostatic hypotension (Table 2).

**TABLE 2:** Cardiovascular risk factors according to manifest orthostatic hypotension in the population

	Orthostatic hypotension		
	Yes	No	*P*
Individuals, *n* (%)	95 (1.7)	5398 (98.3)	N/A
Age	58.4 (4.3)	57.4 (4.3)	0.021
Male sex %	48.4%	46.6%	0.756
BMI	26.9 (3.8)	27 (4.4)	0.686
Weight (kg)	80.3 (15.1)	79.9 (15.7)	0.827
Height (cm)	172.5 (9)	171.6 (9.7)	0.408
WC (cm)	94.7 (11.1)	94.8 (12.7)	0.927
RHR (bpm)	61 (9)	61 (10.6)	0.906
SBP (mmHg)	139 (19)	122 (16)	<0.001
DBP (mmHg)	82 (10)	76 (10)	<0.001
PP (mmHg)	56 (13)	46 (10)	<0.001
Carotid plaque	59.7%	66.3%	0.195
CACS^a^	9 (89)	0 (27)	<0.001
PWV	8.7 (1.4)	8.3 (1.3)	0.004
Pack-years	27 (30)	27 (34)	0.985
Haemoglobin	142 (13)	143 (12)	0.632
Creatinine	78 (14)	77 (15)	0.868
Triglycerides	1.2 (0.8)	1.3 (0.8)	0.541
Cholesterol	5.4 (1)	5.5 (1)	0.629
HDL	1.7 (0.5)	1.7 (0.5)	0.507
LDL	3.5 (0.9)	3.6 (1)	0.441
Chol/HDL	3.5 (1.2)	3.6 (1.3)	0.451
CRP	2.2 (3.6)	2.4 (4.4)	0.774
HbA1c	39 (13)	37 (7)	0.000
Glucose	5.8 (2.1)	5.5 (1.2)	0.035
DM	11.6%	7.6%	0.170
Smoking	10.5%	14.9%	0.306
HTD treatment	25.3%	19.9%	0.417

Values expressed are means (±SD) or percentages. Bpm, beats per minute; CACS, Coronary artery calcification score; Chol, cholesterol; DM, diabetes mellitus; HTD, hypertensive drug; *N*, Total number of participants; PP, pulse pressure; PWV, pulse wave velocity; RHR, resting heart rate; WC, waist circumference.

a Expressed as median and third quartile due to skewed distribution.

In multivariable linear regression models, orthostatic hypotension was associated with PWV (beta 0.27; *P* = 0.023) but not with CACS (*P* = 0.26).

The distribution of cardiovascular risk factors in relation to OH90 is shown in Supplementary Table 1. Of note, individuals with normal orthostatic reactions demonstrated higher values of variables associated with obesity, that is they had higher BMI (*P* = 0.007), weight (*P* = 0.035) and waist circumference (*P* = 0.004). The association with haemodynamic parameters according to OH90 was similar to that of orthostatic hypotension, with the exception of PWV, which did not associate with OH90 (*P* = 0.375).

### Orthostatic blood pressure reactions and cardiovascular risk factors

The second (Q2) and third (Q3) quartiles of systolic orthostatic BP reaction included normal orthostatic BP reactions, whereas Q1 indicates an increase in SBP during orthostatic provocation (more than 10 mmHg) and Q4 include a decrease in SBP during orthostatic provocation of 3 mmHg and more, that is including orthostatic hypotension.

**FIGURE 3:**
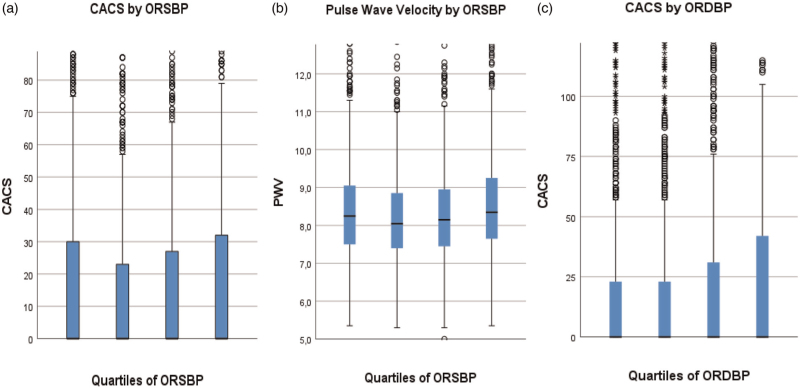
(a--c) Prevalence of coronary calcification and arterial stiffness according to quartiles of orthostatic blood pressure reactions. Coronary artery calcification score (a) and arterial stiffness (b) according to quartiles of orthostatic SBP reaction and coronary artery calcification score according to quartiles of orthostatic DBP reaction (c). Quartiles for orthostatic SBP reaction are shown in Table 1. Quartiles for orthostatic DBP reaction were Q1: increase of 13.5 to 38.5 mmHg; Q2: increase of 9.5 to 13 mmHg; Q3: increase of 5.5 to 9 mmHg; Q4: decrease 26.5 mmHg to increase 5 mmHg. CACS, coronary artery calcification score; ORDBP, orthostatic DBP reaction; ORSBP, orthostatic SBP reaction.

Orthostatic SBP and DBP reactions were associated with CACS (*P* = 0.045 and *P* < 0.001, respectively) as were PP (*P* < 0.001). There was an association between CACS and PWV (*P* < 0.001) (see Figures 3a-c and Supplementary Figure 2A, respectively).

A number of cardiovascular risk factors were distributed according to systolic orthostatic BP reaction in a U-shaped pattern (see Table 1; Fig. 3 and Supplementary Figure 2), that is they were most pronounced in the quartiles with either the most pronounced orthostatic BP increase (Q1) or decrease (Q4). A significant difference across quartiles of systolic orthostatic BP reactions was found for age (*P* < 0.001), CACS (*P* = 0.045) and PWV (*P* < 0.001) (Fig. 3 a,b). The occurrence of carotid plaque differed according to quartiles of orthostatic SBP reactions (*P* = 0.012); however, this was not the case for diabetes mellitus (*P* = 0.079).

For anthropometric measurements, there was an association between orthostatic BP and BMI (*P* < 0.001) and waist circumference (*P* = 0.019), where specifically an increase in orthostatic BP seemed to correlate with higher BMI and waist circumference.

Height was associated with orthostatic SBP decrease (*P* < 0.001), with the largest heights seen in the quartile with the most pronounced orthostatic BP decrease.

Higher SBP, DBP and larger PP (*P* < 0.001 for all) were observed in the quartile with the most pronounced systolic orthostatic BP decrease.

### Resting heart rate and cardiovascular risk factors

Resting heart rate was divided into quartiles Q1 to Q4: less than 56, 56–60, 61–66 and more than 66 bpm, respectively.

**FIGURE 4:**
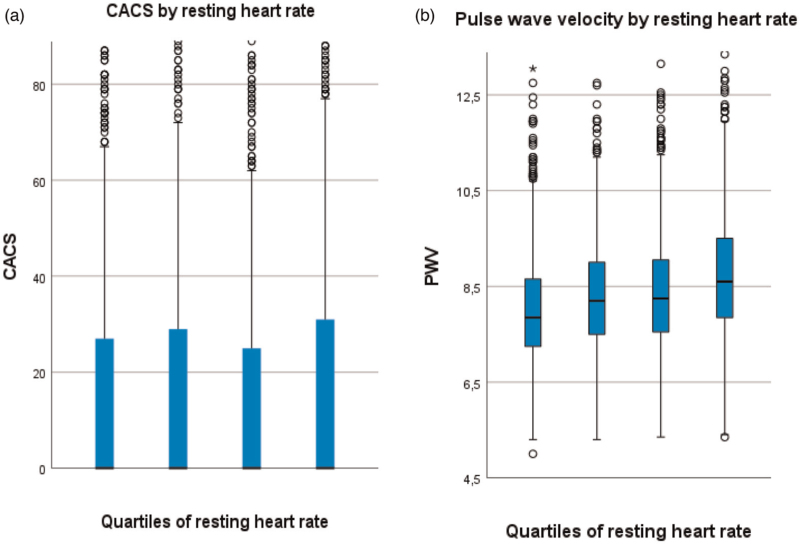
(a,b) Coronary artery calcification and arterial stiffness according to quartiles of resting heart rate. Coronary artery calcification score (a) and arterial stiffness (b) according to quartiles of resting heart rate. Quartiles for resting heart rate were Q1: <56 bpm; Q2: 56–60 bpm; Q3: 61–66 bpm and Q4: >66 bpm. CACS, coronary artery calcification score.

There were no significant differences in CACS according to quartiles of RHR (*P* = 0.137). Visually, an S-formed pattern was observed as depicted in Fig. 4a.

Significant differences according to RHR were found for PWV (*P* < 0.001), age (*P* = 0.011), SBP (*P* < 0.001) and DBP (*P* < 0.001), and the anthropometric parameters BMI (*P* < 0.001), weight (*P* < 0.001), height (*P* < 0.001), waist circumference (*P* < 0.001). A number of biochemical values differed as well, including creatinine (*P* < 0.001), triglycerides (*P* < 0.001), HDL (*P* < 0.001), cholesterol (*P* = 0.026), blood glucose levels (*P* < 0.001), CRP (*P* < 0.001) and HbA1c (*P* < 0.001).

There was no significant association between RHR and PP (*P* = 0.132) and LDL (*P* = 0.089).

For the haemodynamic parameters, the lowest mean SBP and DBP were observed in the quarter with lowest RHR and highest SBP and DBP in the quarter with highest RHR (see Supplementary Figure 2B).

The same pattern was seen for PWV (Fig. 4b) and the anthropometric parameters weight, BMI and waist circumference (Supplementary Figure 2C), except for height, where the pattern was inverted (the quarter with the lowest RHR were on average taller, and the quarter with the highest RHR were on average shorter (Supplementary Figure 2D).

The use of antihypertensive drug treatment was higher in those with higher RHR (*P* < 0.001). The same pattern was seen for the prevalence of diabetes mellitus (*P* < 0.001). Smoking status did not differ according to RHR (*P* = 0.358).

## DISCUSSION

In this study, we tested the hypothesis that an abnormal orthostatic BP response as well as elevated RHR in middle-aged individuals are associated with subclinical cardiovascular disease in the form of increased arterial stiffness, carotid and coronary atherosclerosis. We show that:

(1) The prevalence of manifest orthostatic hypotension is low in the general population at middle age; however, a number of conventional cardiovascular risk factors are associated with this condition already at this age.
(2) There seems to be a U-shaped association between BP reaction upon standing and markers of atherosclerosis and vascular status, including coronary calcium deposits and carotid-femoral PWV. Both an excessive BP increase and decrease upon standing seem to be associated with markers of vascular aging.

The prevalence of orthostatic hypotension and other potential manifestations of cardiovascular autonomic dysfunction increases with age, in parallel with cardiopulmonary disease. Previous studies have shown that orthostatic hypotension as well as other features of cardiovascular autonomic dysfunction are associated with adverse cardiovascular disease outcomes [2,7,24,25]; however, the association between subtle markers of cardiovascular autonomic dysfunction and subclinical cardiovascular disease and risk factors in the general population has been less well explored.

### Generalization of the findings from the current study population

The SCAPIS study enrolled a substantial part of urban age groups 50–64 years of age, with a participation rate over 50%. Hence, findings should be representative for the middle-aged general population in Sweden. Over 20% were obese and more than 7% had DM.

As mentioned, the prevalence of manifest orthostatic hypotension was found to be quite low in our study compared with a similar Swedish study population (The Malmö Preventive Project) that was enrolled some decades earlier [5]. It may be that better control of cardiovascular risk factors in recent decades, with smoking as the most notable difference (45 vs. 15% in our current study), has actually improved vascular health, and thus reduced the prevalence of orthostatic hypotension.

### Cardiovascular disease risk factors associated with cardiovascular autonomic dysfunction

Firstly, we found that a number of cardiovascular risk factors, including age, SBP and DBP, PP, coronary calcium, PWV as well as HbA1c and blood glucose levels were associated with manifest orthostatic hypotension. In contrast, we did not find any significant association between orthostatic hypotension and diabetes, antihypertensive drug treatment and current smoking.

Secondly, we found an inverse correlation between orthostatic hypotension and anthropometric parameters, that is BMI, weight, waist circumference when including SBP drop below 90 mmHg upon standing in the definition of orthostatic hypotension.

Thirdly, we observed U-shaped patterns, that is age, coronary calcium and PWV was higher in those with either excessive BP increase or decrease upon standing, whereas there was a more linear association between calcification and diastolic orthostatic BP decrease.

In agreement with previous studies [1,3,5,9,26], higher BP at rest was strongly associated with manifest orthostatic hypotension, a phenomenon that can be explained by a dysfunction of haemodynamic responses, in turn indicating cardiovascular autonomic dysfunction.

As for the U-shaped pattern between coronary calcium, PWV and orthostatic BP reactions, this is in concordance with recent data indicating that not just orthostatic hypotension but also orthostatic HYPERtension is a considerable risk factor for cardiovascular disease [26]. Subtle signs of autonomic dysfunction, including orthostatic HYPERtension in young individuals, could potentially be a predictor of future hypertension as has been suggested elsewhere [26].

Increased levels of HbA1c and fasting glucose levels associated with manifest orthostatic hypotension in our current study population, whereas there was only a numerical indication of diabetes being more common in orthostatic hypotension. Increased blood glucose levels and high levels of blood lipids over time damage nerves and the small blood vessels that nourish nerves, leading to peripheral autonomic neuropathy [10,27].

Interestingly, the anthropometric parameters BMI and waist circumference were inversely associated with significant orthostatic BP decreases (i.e. either a decrease in SBP of at least 20 mmHg and/or a decrease of DBP of at least 10 mmHg or an absolute SBP < 90 mmHg on orthostatic provocation). In contrast, there was a significant association between height and more pronounced orthostatic SBP decrease. This suggests that overweight and height have opposite effects on the orthostatic BP reaction.

Calcification of the circulatory system is usually progressing with age and leads to hypertension and arterial stiffness. Its rate of progression is affected by cardiovascular risk factors and calcification is a well known pathogenesis behind cardiovascular and circulatory disease [2,5,10,13–16,21]. Calcification at a certain level will lead to arterial stiffness, and as expected, we found a strong association between PWV and manifest orthostatic hypotension. It could not be assessed from the current study whether increased calcification and arterial stiffness (i.e. increased CACS and PWV) leads to abnormal orthostatic BP decrease or increase or whether the association goes the other way around, even if the latter may seem less likely from a pathophysiological point of view. Furthermore, it may be that certain risk factors for vascular ageing lead to the development of both increased arterial stiffness and a propensity towards abnormal orthostatic BP reactions. Further follow-up studies of SCAPIS may shed light on this question.

In discordance with our hypothesis, in this study, we did not find any significant association between RHR and coronary calcium deposits. Our study population was quite healthy and a correlation between elevated RHR and coronary calcium may be found in more severe cases of autonomic dysfunction, for example in older patients with multiple severe morbidities. On the contrary, RHR was associated with a number of additional cardiovascular risk factors, including PWV, which may possibly represent an earlier phenotype of vascular ageing.

In summary, our current findings help us to further understand the haemodynamics in progressing cardiovascular disease [28,29].

### Potential clinical implications

There are some potential clinical implications from our study, mainly from the perspective of CVD prevention and risk assessment. We confirm previous results of manifest orthostatic hypotension being strongly associated with cardiovascular risk factors [1,2,5,6,19,23,24] and that orthostatic hypotension should be regarded as a risk factor that should direct special attention to individuals with overweight, diabetes, hypertension and smoking. Moreover, our results indicate that even subtle deviances in cardiovascular autonomic control, both excessive orthostatic BP increase and decrease as well as RHR, are associated with worse vascular status and higher cardiovascular risk factor burden. Of note, our result further adds to the data that orthostatic hypertension should be regarded as a risk factor for cardiovascular disease, in addition to orthostatic hypotension [26].

### Limitations

Our findings are from the general population of 50–64 years of age, and may not be generalized to other age groups. Atherosclerosis progresses and the prevalence of orthostatic hypotension increases with age; however, the first cardiovascular symptoms such as hypertension typically start to increase in this specific age group [1–3,5].

We performed several tests in this study, and whereas Bonferroni correction may be too conservative due to the high co-relationship between some of the tested variables, individual significant associations for variables should be interpreted with some caution. Still, the overall pattern of our results does indeed indicate an increased risk factor burden and subclinical atherosclerosis in individuals with abnormal orthostatic BP reactions and higher RHR.

As is the nature of observational studies, there has to be considered a potential ‘healthy volunteer’ bias, as low socioeconomic status is associated with a lower participation rate. The prevalence of certain important risk factors in this cohort such as smoking were however comparable (smoking prevalence in Sweden 2018 was around 14%) [18,30].

The cross-sectional design limits the interpretation of the results: We cannot comment on impact on cause and effect between atherosclerosis and arterial stiffness and indices of autonomic cardiovascular dysfunction, but solely that an association exists. Atherosclerosis is a known risk factor of autonomic cardiovascular dysfunction [2,3,5]. However, recent studies suggest that even subtle signs of autonomic cardiovascular dysfunction in young individuals could potentially predict future cardiovascular mortality [26].

Arterial stiffness was determined by carotid-femoral PWV, which is considered the gold standard [11,18]. Orthostatic BP reactions were assessed in a simple way according to guidelines (3 min after standing up) [31]. A more advanced but very resource-intensive assessment of orthostatic BP reactions could be achieved by a tilt-test with prolonged continuous BP and heart rate monitoring in up to 20 min after tilting. An advantage of using the normal simpler protocol is its availability and possible easy implementation in screening and risk assessment in primary care. Coronary artery calcification scoring by low-dose CT is a good way to screen for subtle asymptomatic coronary calcification [12–15,17,21,32]. The score is solely referring to the total degree of calcification and is therefore a good choice in our study to assess even subtle atherosclerosis. However, the score does not add information on the location or severity of possible dangerous plaques. Recent studies even suggest that CTA, which may also detect noncalcified plaque, is better at detecting even more subtle atherosclerosis compared with CACS with the standard CT protocols [20,33].

In conclusion, this study confirms the previously known association between orthostatic hypotension and an increased cardiovascular risk factor burden. In addition, we found that even subtle abnormalities in cardiovascular autonomic control, manifested as either excessive BP increase or decrease upon standing as well as RHR, are associated with a higher cardiovascular risk factor burden in the general middle-aged population.

## ACKNOWLEDGEMENTS

The main funding body of The Swedish CArdioPulmonary bioImage Study (SCAPIS) is the Swedish Heart-Lung Foundation. The study is also funded by the Knut and Alice Wallenberg Foundation, the Swedish Research Council and VINNOVA (Sweden's Innovation agency) the University of Gothenburg and Sahlgrenska University Hospital, Karolinska Institutet and Stockholm county council, Linköping University and University Hospital, Lund University and Skåne University Hospital, Umeå University and University Hospital, Uppsala University and University Hospital. G.E. was supported by the Heart and Lung foundation, the Swedish Research Council, and the Skåne University hospital at the ALF (Avtal om Läkarutbildning och Forskning) agreement. V.H. was supported by the Swedish Heart and Lung foundation, the Medical Faculty of Lund University, Governmental funding within the Swedish National Health Services, Skåne University Hospital Funds, Crafoord Foundation, Ernhold Lundströms Research Foundation, Region Skåne, Hulda and Conrad Mossfelt Foundation and Anna-Lisa and Sven Eric Lundgrens Foundation for Medical Research.

### Conflicts of interest

S.G. is employed by Novo Nordisk.

## Supplementary Material

Supplemental Digital Content
