# Supplementary material for: Subclinical atherosclerosis and risk factors in relation to autonomic indices in the general population
Source: J Hypertens. 2023 Mar 20;41(5):759–67. doi: 10.1097/HJH.0000000000003397 (PMC10090316; doi:10.1097/HJH.0000000000003397)

**Supplementary Table 1**

|  | **OH including SBP < 90 mmHg** | | ***P*** |
| --- | --- | --- | --- |
|  | **Yes** | **No** |  |
| **Age** | 58 (4.4) | 57.4 (4.3) | 0.149 |
| **Male sex %** | 40.3 % | 46.8 % | 0.172 |
| **BMI** | 26 (3.9) | 27 (4.4) | 0.007 |
| **Weight (kg)** | 77 (15.4) | 80 (15.7) | 0.035 |
| **Height (cm)** | 171.6 (9.1) | 171.6 (9.7) | 0.985 |
| **WC (cm)** | 91.5 (12) | 94.9 ( 12.7) | 0.004 |
| **RHR (bpm)** | 60 (10) | 61 (9) | 0.567 |
| **SBP (mmHg)** | 128 (26) | 123 ( 16) | 0.001 |
| **DBP (mmHg)** | 77 (14) | 76 (10) | 0.816 |
| **PP (mmHg)** | 51 (15) | 46 (10) | 0.000 |
| **CACS ^a^** | 2 (57) | 0 (27) | 0.022 |
| **PWV** | 8.2 (1.5) | 8.3 (1.3) | 0.375 |
| **Pack-years** | 28 (31) | 27 (34) | 0.902 |
| **Hemoglobin** | 140 (13) | 143 (12) | 0.037 |
| **Creatinine** | 76 (14) | 77 (15) | 0.235 |
| **Triglycerides** | 1.1 (0.7) | 1.3 (0.8) | 0.052 |
| **Cholesterol** | 5.4 (1) | 5.5 (1) | 0.260 |
| **HDL** | 1.8 (0.6) | 1.7 (0.5) | 0.079 |
| **LDL** | 3.5 (0.9) | 3.6 (0.9) | 0.141 |
| **Chol/HDL** | 3.3 (1.2) | 3.6 (1.3) | 0.043 |
| **CRP** | 1.9 (3.2) | 2.4 (4.4) | 0.278 |
| **HbA1c** | 38 (12) | 37 (7) | 0.022 |
| **Glucose** | 5.7 (1.9) | 5.5 (1.2) | 0.262 |
| **DM** | 7.7 % | 8.5 % | 0.393 |
| **Smoking** | 10.5 % | 14.9 % | 0.201 |
| **HTD treatment** | 19.4 % | 20 % | 0.915 |
| **Legend:**  Values expressed are means (±SD) or percentages.  ^a^ expressed as median and 3^rd^ quartile due to skewed distribution.  OH: Orthostatic hypotension; WC: Waist circumference; RHR: Resting heart rate; Bpm: Beats per minute; SBP: Systolic blood pressure; DBP: Diastolic blood pressure; PP: Pulse pressure; CACS: Coronary artery calcification score; PWV: Pulse wave velocity; Chol: Cholesterol; DM: Diabetes; HTD: Hypertensive drug. | | | |

**Supplementary figure 1:** The distribution of body mass index in the study population.


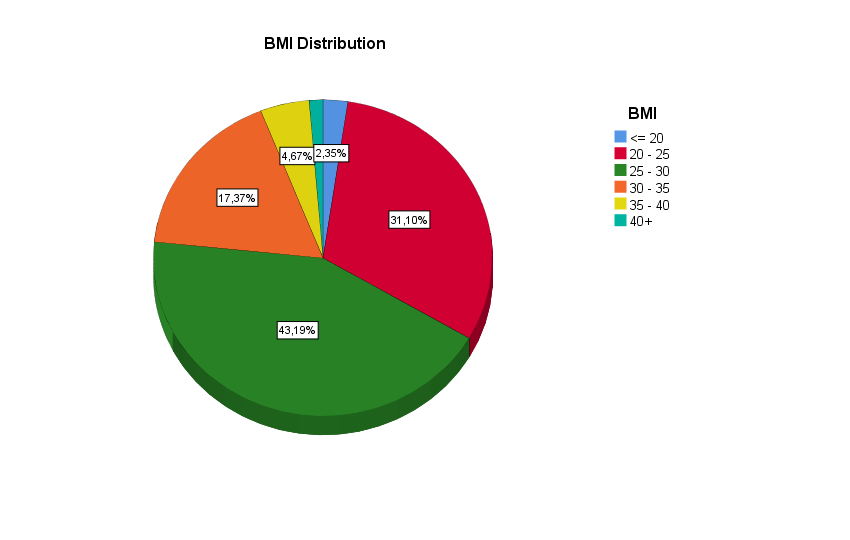


**Supplementary figures 2A-D:** Relations between hemodynamic parameters and cardiovascular risk factors.


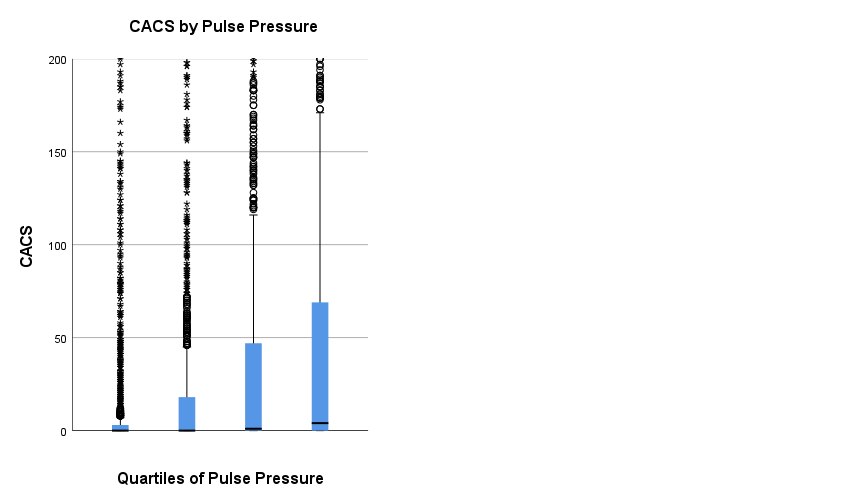

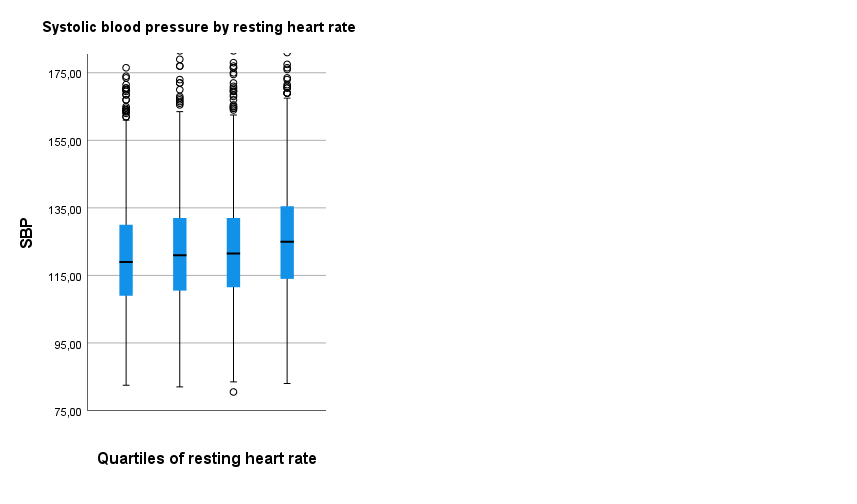

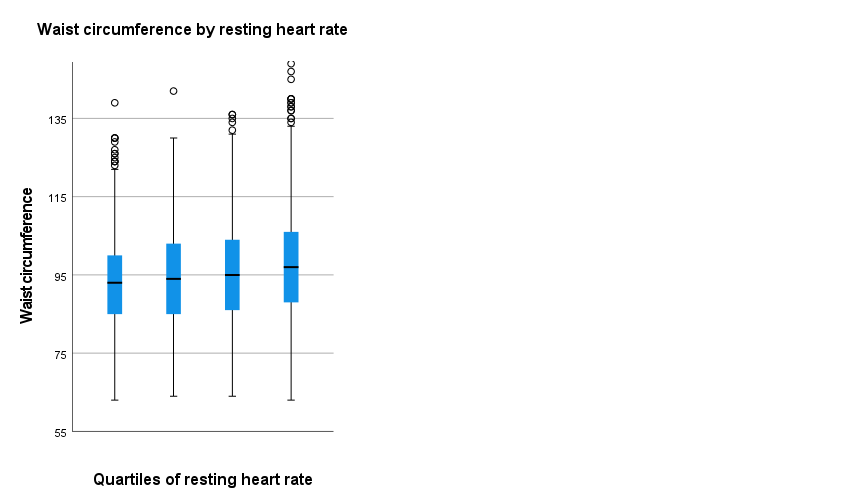

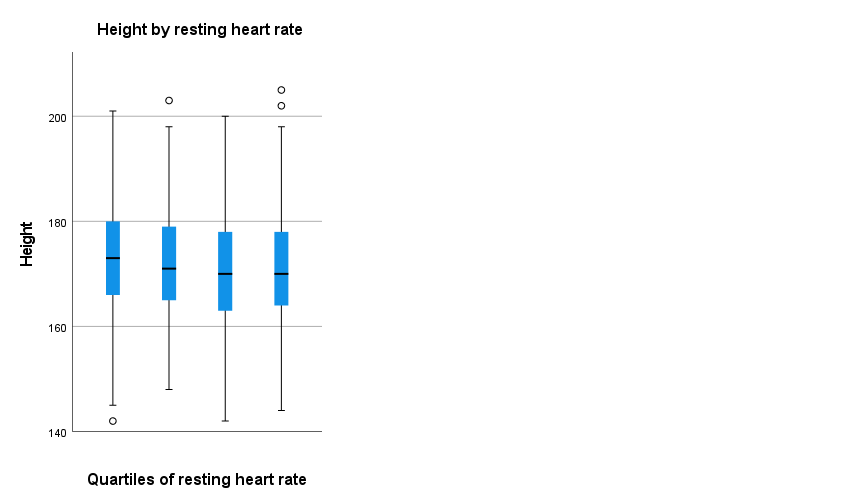

Supplement: Supplemental Digital Content [file jhype-41-759-s001.docx]
